# Comments on Quaak, et al. The Dynamics of Autism Spectrum Disorders: How Neurotoxic Compounds and Neurotransmitters Interact. *Int. J. Environ. Res. Public Health* 2013, *10*, 3384–3408

**DOI:** 10.3390/ijerph13121207

**Published:** 2016-12-03

**Authors:** Moslem Mohammadi

**Affiliations:** Molecular and Cell Biology Research Center, Department of Physiology and Pharmacology, School of Medicine, Mazandaran University of Medical Sciences, Sari 48471-91971, Iran; mohammadimo@yahoo.com; Tel.: +98-1133543081

**Keywords:** paraoxon, synaptosome, uptake

## Abstract

I have read the article entitled “The dynamics of autism spectrum disorders: how neurotoxic compounds and neurotransmitters interact”. There are some errors in the interpretation of results obtained from our previous studies that should be explained.

It was with great interest that I read the nice article by Quaak et al. entitled “The dynamics of autism spectrum disorders: how neurotoxic compounds and neurotransmitters interact” published in the International Journal of Environmental Research and Public Health [1] and thank them for having cited some of our papers. Although the authors present findings of our studies published in 2007 [2] and 2008 [3], there are some errors in the interpretation of our results that should be explained as follows:

1. The authors stated “in a study by Ghasemi et al., male Wistar rats were exposed for 20 min to either nanomolar (10^−9^–10^−7^) or micromolar (10^−6^–10^−3^) doses of paraoxon. After exposure to nanomolar doses of paraoxon the authors reported significantly increased GABA uptake. In contrast, after exposure to micromolar doses of paraoxon, a significant reduction of GABA uptake was reported”. In the in vitro study conducted by Ghasemi et al. [2], synaptosomes were prepared from cerebral cortex of untreated rats. After 20 min incubation of synaptosomes with different concentrations of paraoxon (Sigma Chemical Co., Schnelldorf, Germany), GABA uptake was determined. The results showed that paraoxon at low concentrations (10^−9^–10^−6^ M) increased and at higher concentrations (10^−5^–10^−3^ M) decreased the GABA uptake.

2. The authors indicated “Mohammadi et al. reported a significant decrease of GABA uptake by synaptosomes derived from the hippocampus and cortex of adult male Wistar rats after exposure to 0.1, 0.3 and 0.7 mg/kg of paraoxon”. In the study conducted by Mohammadi et al. [3], male Wistar rats were given a single intraperitoneal injection of one of three doses of paraoxon (0.1, 0.3, or 0.7 mg/kg) and [^3^H]GABA uptake was measured in synaptosomes prepared from cerebral cortex and hippocampus. The results showed that the GABA uptake was significantly reduced by both cerebral and hippocampal synaptosomes, only in animals treated with the convulsive dose (0.7 mg/kg) of paraoxon.
